# Prevalence of HBsAg and knowledge about hepatitis B in pregnancy in the Buea Health District, Cameroon: a cross-sectional study

**DOI:** 10.1186/1756-0500-7-394

**Published:** 2014-06-25

**Authors:** Andreas A Besong Frambo, Julius Atashili, Peter Nde Fon, Peter Martins Ndumbe

**Affiliations:** 1 Department of Public Health and Hygiene, Faculty of Health Sciences, University of Buea, P.O, Box, 63, Buea, Cameroon

**Keywords:** Hepatitis B virus, Prevalence, HBsAg, Pregnancy, Cameroon, Knowledge

## Abstract

**Background:**

Although infection with Hepatitis B Virus (HBV) remains a global public health problem, little is known about its epidemiology in pregnancy in sub-Saharan Africa. This study sought to determine the prevalence of, and identify factors associated with hepatitis B surface antigen (HBsAg) positivity among pregnant women in the Buea Health District (BHD) in rural Cameroon. We also assessed pregnant women’s knowledge about hepatitis B.

**Methods:**

A cross-sectional, descriptive study was undertaken. Participants were evaluated using a structured questionnaire with clinical examination and were then screened for HBsAg using a commercial rapid diagnostic test. Assessment of knowledge was done using a hepatitis B basic knowledge summary score.

**Results:**

Of the 176 pregnant women studied, 9.7% (95% CI: 5.7%, 15%) tested positive for HBsAg. None of the risk factors assessed was significantly associated with HBsAg positivity. The hepatitis B knowledge summary score ranged from 0 to 12 with a mean of 1.5 (SD = 3.14, median = 0, IQR = 0 to 0). Only 16% of participants had scores greater than 6/12. The knowledge summary score of the participants was associated with the educational level (p-value = 0.0037).

**Conclusion:**

The high prevalence of HBsAg (9.7%) among women of child bearing age suggests that vertical transmission of HBV may be a public health problem in Buea Health District. Knowledge of HBV among pregnant women was poor. We recommend that all pregnant women ought to be routinely screened for HBV and that health education on HBV should be provided to pregnant women especially during antenatal visits.

## Background

Infection with the hepatitis B virus (HBV) occurs worldwide and constitutes a major public health problem [1]. Over 380 million people worldwide are chronic carriers of HBV and more than 2 million deaths occur annually from HBV related diseases [2]. Approximately 65 million of all chronically infected individuals live in Africa [3,4]. In Cameroon, recent studies reported HBV prevalence rates as high as 10.1% and 12.1% among blood donors in hospital blood banks [5,6]. The prevalence of HBsAg in pregnancy does not vary much from that in the general population [7]. In Cameroon, studies carried out by Ndumbe *et al.* in 1992 and Chiaramonte *et al.* in 1991 revealed that the prevalence of hepatitis B surface antigen was greater than 8% [8,9]. Two more recent studies in Yaounde, Cameroon, estimated the prevalence of HBsAg in pregnancy at 7.9% (in 2005) and 7.7% (in 2013) [10,11]. Little is known on the prevalence of hepatitis in pregnancy in non-urban areas.

The risk factors for hepatitis B infection are known to be linked to body fluids especially those with high concentration of the virus like blood, semen and vaginal secretions [12]. Traditional practices that expose people to hepatitis B infection like scarification, ear and nose piercing as well as tattoos have led to higher prevalence in certain zones but not necessarily in pregnancy [13]. The risk factors for hepatitis B infection during pregnancy vary among communities depending on the cultural practices and some traditional beliefs. In one study carried out in a Nigerian obstetric population the major risk factors identified were higher mean parity, higher number of sexual partners since sexual debut, polygamy and previous sexually transmitted infection [14]. In addition to determining prevalence, risk factors for HBV infection need to be identified in each setting in order to potentially design targeted preventive measures.

Hepatitis B virus (HBV) infection may go undetected. Unawareness of an ongoing infection delays the diagnosis of HBV-related liver disease and favors the spread of the virus [15]. In a study to assess awareness of HBV infection among patients in a clinic in Italy, it was noticed that up to 40.3% of the participants were not aware of their infection [16]. Studies to assess the knowledge of hepatitis B are more often conducted with health personnel being participants. Pregnant women too are a vulnerable group and theirs is the risk of transmitting the infection to the newborn if the mother is infectious. It is for these reasons that this study was designed to determine the prevalence of HBsAg among pregnant women in the BHD and to identify the risk factors associated with hepatitis B in pregnancy. The knowledge of participants about hepatitis B was also assessed in this study.

## Methods

This cross-sectional, descriptive study was carried out in antenatal clinics (ANC) in the Buea Health District of Cameroon from the June to July 2012. The ANCs involved were those of the Buea Regional Hospital, Buea Road Health Center, Buea Town Health Center, Muea Subdivisional Hospital, Mile 16 Health Center and Solidarity clinic. There are other health centers found in the health district, but because of their low number of pregnant women per ANC session (<4 per week) and the irregularity of their ANC sessions, they were not included in our study setting. Our study and target population was made up of pregnant women who attend ANC in the health district. The sampling method was consecutive. All pregnant women attending ANC in the health district were eligible for participation and were included if they agreed to participate. All participants who consented were interviewed using a standard questionnaire adapted from the WHO protocol for assessment of hepatitis B infection in antenatal patients [17]. Prior to use in the study participants the questionnaire was pilot tested in 16 pregnant women in our setting with the aim of revising poorly structured questions, estimate the average time required to fill the questionnaire and thus validate the use of the questionnaire in our context.

After the examination of the participant, 3 mls of fresh venous blood was collected by venipuncture into an EDTA tube. The plasma of collected samples was tested for presence of HBsAg using a commercial hepatitis B surface antigen test strip, the DiaSpot One Step Hepatitis B test (DiaSpot Rapid Diagnosis, Jakarta, Indonesia). This required the deposition of two drops of the plasma sample onto the corresponding receiving section of the strip and waiting a minimum of five minutes before reading the test result. The test result is said to be positive when two bands appear; one in the test region and another in the control region. The test result was marked negative when one band appears in the control region and no band appears in the test region. In situations where the control region had no test band, the test was marked invalid. For invalid cases, the used strip is discarded. A new strip is gotten and the remaining blood in the EDTA test tube was used to obtain another identical sample. This was again tested in a similar manner and the result obtained recorded accordingly.

### Statistical analysis

The analysis of the data was done using EPI-INFO Version 3.5.1 and MICROSOFT EXCEL-2007. The prevalence of HBsAg among the participants was the ratio between HBsAg positive samples and the total samples collected. For every potential risk factor assessed (history of surgery, history of blood transfusion, abortion, scarification, piercing, tattoos, condom use, sex partners and history of sexually transmitted infection), the risk ratio of antigen positivity comparing those with potential risk factor to those without the risk factor was determined. The statistical significance of the association between each potential risk factor and antigen positivity was assessed using a Fisher’s exact test. Risk factors with p-values < 0.05 were to be considered significant and associated. The knowledge of hepatitis B was assessed by determining how many participants had correct responses to the questions on knowledge of hepatitis B. Each knowledge question was scored as zero (0) for incorrectly or “Don’t know” responses, while correct responses were scored one (1). A knowledge summary score for the 12 questions asked was calculated from the total of correct answers. Participants with scores over 6/12 were denoted knowledgeable. Data are presented using tables.

### Ethical considerations

Ethical clearance was obtained from the Faculty of Health Sciences Institutional Review Board (FHS-IRB) of the University of Buea and administrative authorization was obtained from the Regional Delegation of Public Health for the South West Region of Cameroon. Participants had the study protocol carefully explained to them and participation was voluntary. Written informed consent was obtained from all participants. All procedures were standard involving minimum risks. Study results were returned to participants and incorporated into their care by their respective providers.

## Results

### Participant characteristics

The characteristics of the 176 participants enrolled are summarized in Table 1. The ages ranged from 17 to 42 years with a mean age of 25.7 ± 5.4 years. The predominant age group was 25–29 years representing 33% of the general population. The majority of the participants had completed secondary school. Thirty-six (20.7%) of the participants were students and a greater part of the participants 94 (54%) were employed. Amongst the married participants, 108 (62.1%) were monogamous while 5 (2.9%) were polygamous. Regarding gestational age and parity, majority of the participants were in the third trimester (64%) and multigravidas comprised the majority (58.4%) when compared with primigravidas (31.8%) and grand multigravidas (9.8%).

**Table 1:** Demographic characteristics of participants of a survey of HBsAg prevalence among pregnant women in Buea Health District

**Characteristic**	N*	**Percentage**
AGE (years)		
< 30	139	79
≥ 30	37	21
Education		
Informal	1	0.6
Primary	33	19.3
Secondary	89	52.0
Tertiary	48	28.1
Employment		
Unemployed	44	25.3
Employed	94	54.0
Student	36	20.7
Marital Status		
Unmarried	61	35.1
Monogamy	108	62.1
Polygamy	5	2.9
Gestational age		
First trimester	10	6.1
Second trimester	49	29.9
Third trimester	105	64.0
Parity		
Primipara (1)	55	31.8
Multipara (2–4)	101	58.4
Grand multipara (>4)	17	9.8

*Some item totals may not sum up to 176 because of non-response.

### Prevalence of HBsAg

The prevalence of HBsAg in pregnancy was found to be 9.7% (95% CI, 5.7 – 15.0). The highest prevalence rate was observed in the age group 15–19 (20%), followed by the age group 30–34 (13.64%). None of the assessed participant characteristics was significantly associated with HBsAg positivity (Table 2). Half of the participants with HBsAg positivity were in the third trimester and multigravidas (64.7%) constituted the majority of HBsAg positive women. Twenty participants had right upper quadrant tenderness among whom 4 (20%) were positive for HBsAg. No other assessed clinical feature (hepatomegaly, jaundice, palmar erythema, splenomegaly, finger clubbing, abnormal urine and stool colour) suggestive of liver disease was identified among the participants that tested positive for HBsAg.

**Table 2:** Prevalence of HBsAg in relation to socio-demographic and obstetrical characteristics of pregnant women attending ANC in the Buea Health District, Cameroon

**Factor**	**N**	**n**	**%**	**RR**	**Fisher exact p-value**
Age				0.81	1.00
< 30	139	14	10.07		
≥ 30	37	3	8.11		
Education				0.74	0.53
≤ Primary	34	4	11.76		
>Primary	137	12	8.76		
Employment				1.22	0.80
Unemployed	80	7	8.75		
Employed	94	10	10.64		
Marital status				1.75	0.42
Unmarried	61	4	6.56		
Married	113	13	11.50		
Gestational age				0.56	0.27
≤ 24 weeks	59	8	13.56		
>24 weeks	105	8	7.62		
Parity				0	0.38
≤ 4	156	17	10.9		
>4	17	0	0		

N = number of participants tested, n = positives, % = prevalence, RR = risk ratio.

None of the risk factors assessed in this study was significantly associated with HBsAg positivity. Table 3 shows the different risk factors assessed and their corresponding risk ratios and p-values.

**Table 3:** Risk factors associated with HBsAg positivity among pregnant women in Buea Health District

**Risk factor**	**Number**	**Positives**	**Percentage**	**Risk ratio (RR)**	**p-value**
Past surgery				0.00	0.23
No	153	17	11.11		
Yes	21	0	0.00		
Blood transfusion				0.00	0.61
No	161	17	10.56		
Yes	12	0	0.00		
Abortion				0.88	1.00
No	118	12	10.17		
Yes	56	5	8.93		
Scarification				0.84	0.79
No	66	7	10.61		
Yes	101	9	8.91		
Piercing				1.62	1.00
No	16	1	6.25		
Yes	158	16	10.13		
Tattoos				0.00	1.00
No	142	15	10.56		
Yes	4	0	0.00		
Condom use				0.00	1.00
Inconsistent	169	17	10.06		
Consistent	7	0	0.00		
Sex partners				0.71	1.00
≤5	159	16	10.06		
>5	14	1	7.14		
Past sti				0.69	0.46
No	87	10	11.49		
Yes	88	7	7.45		

### Knowledge on hepatitis B

Table 4 shows the responses to questions about knowledge of HBV among the pregnant women who participated in this study. As a whole, majority of the participants had never heard of the disease called hepatitis and 80% of the participants did not know that hepatitis B was a virus. Only 15.9 % of the participants knew that infection with hepatitis virus affects the liver as the primary organ. The knowledge of HBV regarding transmission was equally poor as < 20% of the participants had the correct knowledge. A hundred and forty one (80.6%) among the 176 who participated in this study did not know that an infected person is capable of living without ever presenting complaints related to the disease. Meanwhile, a smaller 2.3% had the misconception that only particular people could be affected by the hepatitis B virus. The fact that hepatitis B infection can cause liver cancer was affirmed by 28 participants (15.9%) and 15.3% had the correct knowledge about prevention of hepatitis B infection by vaccination.

**Table 4:** Knowledge of HBV among pregnant women attending ANC in the Buea Health District, Cameroon

**Knowledge question**	**No (%)**	**Yes (%)**	**Don’t know (%)**
Is hepatitis B a virus?	**5 (2.9)**	**30(17.1)**	**140 (80)**
Does hepatitis B affect the liver?	**5 (2.8)**	**28 (15.9)**	**143 (81.3)**
Can hepatitis B be transmitted through unsterilized needles, blades and other sharp material?	**6 (3.8)**	**22 (12.5)**	**148 (84.1)**
Can hepatitis B be transmitted by contaminated blood and blood products?	**4 (2.3)**	**29 (16.5)**	**143 (81.3)**
Is hepatitis B transmitted through unsafe sex?	**7 (4.0)**	**24 (13.7)**	**144 (82.3)**
Can hepatitis B be transmitted through kissing?	**16(10.9)**	**19 (15.0)**	**140 (80)**
Can an infected person remain without symptoms?	**14 (8.0)**	**20 (11.4)**	**141 (80.6)**
Can hepatitis B affect any person?	**4 (2.3)**	**32 (18.2)**	**140 (79.5)**
Will an infected person remain infected for life?	**18(11.4)**	**10 (8.0)**	**146 (80.6)**
Is a specific diet required for all infected persons?	**12 (6.9)**	**10 (5.7)**	**153 (87.4)**
Can hepatitis B infection cause liver cancer?	**4 (2.3)**	**28 (15.9)**	**144 (81.8)**
Can hepatitis B be prevented by vaccination?	**6 (3.4)**	**27(15.34)**	**143 (81.3)**

Note. Knowledge of HBV was assessed by assigning a point for every correct answer only. Points ranged from 0 to 12 with a mean of 1.5 ± 3.14 (median = 0, IQR = 0 to 0). Scores ≤ 2 were considered inadequate while scores within 3 and 5 were considered intermediate. Scores ≥ 6 were considered adequate and acceptable.

Out of the 176 participants, 137 (80%) had a knowledge summary score ≤ 2 while 7 (4%) had a knowledge summary score that ranged between 3 and 5. Twenty eight (16%) participants had a knowledge summary score ≥ 6 and were denoted knowledgeable for HBV. Both inadequate and intermediate knowledge scores were merged and termed poor knowledge so that a cut-off value for the knowledge summary score of 6 will be considered poor knowledge. In this case 84% of the participants fell under the range of poor knowledge.

Among the demographic and obstetric characteristics, only education was significantly associated with mean KSS (p value = 0.0037).

## Discussion

Screening asymptomatic people is an important instrument of disease detection, prompt diagnosis and intervention especially concerning a typically asymptomatic infection such as chronic HBV infection. Given that infected pregnant women stand the chance of transmitting the HBV infection to their newborn babies, we decided to conduct a cross-sectional study to determine what proportion of pregnant women attending ANC in Buea Health District have been affected by HBV. The information gathered from this piece of work will be used by the health care providers in the health district to make decisions in an attempt to improve on the health standards of the district as a whole, but specifically for pregnant women.

In the present study, the prevalence of HBsAg among pregnant women was found to be 9.7%. This is relatively high in view of the fact that a vast majority of participants were asymptomatic. In comparison with studies from other parts of the same country, the prevalence reported in this study was higher than the 1.2%, 7.85% and 7.7% reported by Thumamo &Asoquo (2004) , Kfutwah *et al.* (2012) and Fomulu *et al.* (2013) respectively [11,18,19]. This difference may be because of different socioeconomic status and the natural difference attached to different geographic zones. Our prevalence was also higher than the 5.4% reported in the early nineties by Ndumbe et al. in rural pregnant women in Cameroon [20]. We had similar results with Okoth *et al.* in Kenya, who found prevalence of 9.3% [21]. Similar results were also recorded by Makuwa *et al.* who found a prevalence of 9.2% in Gabon [22]. These similarities could be explained by the fact that it is the same risk group that was studied and also the test used was similar in principle to the one we used in our study. We however expected this high prevalence and this is in conformity with the established fact that HBsAg is endemic in African countries south of the Sahara desert of which Cameroon is among [8]. This level of carrier state in women of reproductive age will suggest that there is a high risk of mother-to-infant transmission in the Buea Health District. However, HBsAg carrier state alone in a pregnant woman does not put the foetus at a huge risk of being infected, instead, pregnant women who are positive both for HBsAg and HBeAg are more likely to infect their babies. Transmission does not only depend of HBsAg and HBeAg status but also on anti-HBeAg status - anti-HBeAg positivitiy tends to prevent transmission [23-25]. It is estimated that transmission rates can be as high as 12%, 25% and 70-90% respectively in HBeAg negative/anti-HBe positive mothers, HBeAg negative/anti-HBe negative mothers and HBeAg positive/anti-HBe negative mothers [26]. Other studies have suggested that as many as 41% of HBsAg pregnant women are also HBeAg positive [27]. If this were the case in the BHD then the risk of vertical transmission in this setting will still be high. More accurate estimates of the risk of mother-to-infant transmission will however require a study of HBsAg, HBeAg, anti-HBeAg levels and HBV_DNA levels (when possible) in pregnant women in the Buea Health district. Given the unavailability of the hepatitis B immune globulin (HBIG) in many developing countries, the use of the hepatitis B vaccine alone is recommended and considered appropriate treatment to prevent many cases of HBV during the perinatal period – the period of highest risk of chronicity [28].

We did not identify significant risk factors associated with HBsAg positivity in our study. This is contrary to many studies carried out elsewhere as most often at least one risk factor is identified. Blood transfusion was identified as the single risk factor for HBsAg positivity in Mexico by Cisneros-Castolo *et al.*[29]. Rabiu *et al.,* identified three risk factors associated with HBsAg positivity in Nigeria (early age of sexual debut, history of multiple sexual partners and history of sexually transmitted infection) [30]. In a more recent study in urban Cameroon, only a history of contact with a jaundiced or HBV-infected person was identified as a significant predictor of current HBsAg positivity [11]. From these studies, it is evident that different regions have different risk factors associated with HBsAg positivity. Nevertheless, blood transfusion still remains a risk factor for hepatitis B transmission irrespective of the region, due to undetected donors with early acute infection, resolving infection, silent infection or infection with atypical virus serology [31]. The main reason why we think we did not identify risk factors associated was probably because our sample size was small and was statistically determined for prevalence, not for risk factors. Also it could be that the risk factors we assessed are not among the risk factors associated with HBsAg positivity in Buea Health District. Furthermore, because participants could not possibly remember what happened to them at birth and childhood, we could not assess two important risk factors associated with chronic carrier status: being borne of an HBV-positive mother and early contact in childhood with HBV [32,33].

Apart from right upper quadrant tenderness which was reported in one in five HBsAg-positive participants, all other typical symptoms of hepatitis were rare. This is not surprising as it confirms the chronic rather than acute nature of the infection in these women.

Our result document very low levels of knowledge about HBV infection among pregnant women who attend ANC in Buea health district in general. Specifically we noticed that more than 80% of our participants did not know hepatitis B was a disease caused by a virus until it was explained to them. Such low knowledge was also observed in Seattle, Washington, by Taylor and colleagues [34]. Furthermore, well above 80% of our respondents had no knowledge of the different methods of transmission of HBV until it was explained to them in terms of similarity of transmission with HIV with the emphasis on higher infectivity associated with HBV when compared to HIV. This is similar to earlier studies carried out by Sharma *et al.*[35]. The magnitude of awareness regarding HBV transmission has generally been observed to be quite lower than HIV though both have similar modes of transmission, even in studies on different risk groups like barbers and patients on dialysis. This difference in knowledge and precisely the low level of awareness of HBV is probably because of the lack of formal education available about HBV as compared to other diseases of similar modes of transmission and burden. Majority of our respondents did not know that most HBV carriers are asymptomatic and could transmit the virus to an uninfected person through sexual intercourse. The fact that HBV can affect any person was known to most of our participants. Overall the poor knowledge shown in this study is in line with findings from Pakistan [36] and around the globe [34,37]. This deficient knowledge warrants the need for sustained and continuous education not only about HBV but other diseases of similar magnitude and burden.

A number of limitations need to be considered in interpreting these findings. First, only HBsAg was tested as a marker for hepatitis B infection. Other markers exist which if combined with HBsAg could have been more reliable. As such this study only determined the prevalence of HBsAg in pregnancy in our locality. It did not permit the calculation of the risk of mother-to-child HBV transmission. Furthermore considering the cross-sectional design, it is not possible to rule out or infer any cause-effect relationship between the factors assessed and HBsAg positivity. Studying self-reported knowledge is itself a limitation as one cannot rely totally on the information provided by the participants because of recall bias and social desirability bias. The use of a rapid test (less sensitive when compared to ELISA and PCR tests) may also have resulted in an underestimation of the prevalence of HBsAg.

## Conclusion

Hepatitis B virus infection is a public health problem in Buea Health District. A 9.7% prevalence of HBsAg in an apparently healthy population of pregnant women attending ANC was recorded in this study. None of the risk factors assessed was significant for HBsAg positivity. Further studies using a larger sample size will be needed to assess the risk of mother-to-child transmission of HBV in this setting. If these high prevalence rates are confirmed, then the public health policies to reduce the burden of HBV, including universal immunisation, and screening in high risk groups, need to be reinforced. Only 16% of the pregnant women were knowledgeable about HBV. More than 80% had misconceptions regarding the methods of transmission of HBV. While keeping in view the global burden of HBV, it is important to focus on women of child- bearing age and educate them on HBV and its prevention.

## Abbreviations

ANC: Antenatal clinic; BHD: Buea Health District; CI: Confidence interval; EDTA: Ethylenediaminetetraacetic acid; HBIG: Hepatitis B immune globulin; HBsAg: Hepatitis B surface antigen; HBV: Hepatitis B virus; HIV: Human immunodeficiency Virus; KSS: Knowledge summary score; mls: Millilitres; SD: Standard deviation; WHO: World Health Organization.

## Competing interests

The authors declare that they have no competing interests.

## Authors’ contributions

AABF, JA, PNF, and PMN conceived and designed the study. AABF implemented the study. AABF and JA conducted data analysis. AABF, JA, and PNF interpreted study results: AABF wrote the first draft of the manuscript. JA, PNF, and PMN reviewed and corrected the draft manuscript. All authors read and approved the final manuscript.
